# Sodium-Glucose Cotransporter 2 Inhibitors in Diabetic Nephropathy: A Systematic Review and Meta-Analysis

**DOI:** 10.7759/cureus.107083

**Published:** 2026-04-15

**Authors:** Jaisingh Rajput, Prajakta V Rajput, Nihar Jena, Abhishek Kumar Mariswamy Arun Kumar, Elan Mohanty, Saketh Parsi

**Affiliations:** 1 Family Medicine, Montgomery Family Medicine Residency Program, Baptist Health, Montgomery, USA; 2 Hospital Medicine, Baptist Medical Center East, Montgomery, USA; 3 Cardiovascular Medicine, Wayne State University, Detroit, USA; 4 Hospital Medicine, Kettering Health Network, Dayton, USA; 5 Internal Medicine, Providence St. Mary Medical Center, Apple Valley, USA; 6 Hospital Medicine, Ascension Seton Medical Center Austin, Austin, USA

**Keywords:** diabetic nephropathy, meta-analysis, renal insufficiency, sodium-glucose transporter 2 inhibitors, systematic review

## Abstract

One of the most common causes of end-stage renal disease, which still inflicts a significant clinical and economic burden, is diabetic nephropathy. Even though glycemic and blood pressure management have improved, a considerable proportion of patients progressing to renal failure develop progressive renal dysfunction. Sodium-glucose cotransporter 2 (SGLT2) inhibitors have also proven to be agents with far-reaching renoprotective effects beyond glucose lowering.

The present systematic review and meta-analysis synthesizes the existing evidence on the effectiveness and safety of SGLT2 inhibitors in diabetic nephropathy. The review of randomized controlled trials was conducted in line with PRISMA requirements. Twenty studies comprising 131,011 participants were included.

SGLT2 inhibitor therapy significantly reduced the risk of composite renal outcomes by 30% (pooled relative risk, 0.70; 95% confidence interval, 0.67 to 0.73; p < 0.001), with minimal heterogeneity across studies (I² = 8.3%). The benefit was consistent across all SGLT2 inhibitor classes (p-value for between-group difference = 0.682), across all stages of chronic kidney disease (CKD) from early to advanced (p = 0.658), and regardless of background renin-angiotensin system (RAS) inhibitor use (p = 0.795).

This meta-analysis provides robust evidence that SGLT2 inhibitors confer significant renal protection in patients with diabetic nephropathy, with consistent benefits observed across all drug classes, across all stages of CKD, and irrespective of background RAS inhibitor use. These findings support the incorporation of SGLT2 inhibitors as foundational organ-protective therapy in contemporary clinical practice guidelines for diabetic kidney disease management.

## Introduction and background

Diabetic nephropathy is a significant microvascular complication of diabetes mellitus, and it is the leading cause of end-stage renal disease in different parts of the world [1]. The syndrome is typified by chronic albuminuria, a gradual decline in glomerular filtration rate, and high blood pressure, and it has a close correlation with greater cardiovascular morbidity and mortality [2]. Despite the reported advances in glucose control and renin-angiotensin system (RAS) blockade leading to a prolongation of disease progression, a significant fraction of patients still progress to kidney failure [3].

The initial development of sodium-glucose cotransporter 2 (SGLT2) inhibitors was as antihyperglycemic agents, and their effect was to inhibit glucose reabsorption in the proximal renal tubule. Nevertheless, the results of large cardiovascular and renal outcome trials have shown significant advantages that cannot be reduced solely to glycemic control [4,5].

The renoprotective mechanisms of SGLT2 inhibitors extend beyond glycemic control and are primarily hemodynamic. By inhibiting sodium and glucose reabsorption in the proximal convoluted tubule, these agents increase sodium delivery to the macula densa, activating tubuloglomerular feedback. This physiological response triggers afferent arteriolar vasoconstriction, which reduces intraglomerular hypertension and hyperfiltration, the key drivers of diabetic kidney disease progression [6]. Additionally, SGLT2 inhibitors decrease the activity of the intrarenal renin-angiotensin-aldosterone system, reduce renal oxygen demand, and improve mitochondrial function, thereby attenuating hypoxic injury and fibrosis [7,8]. These hemodynamic effects explain the immediate reduction in estimated glomerular filtration rate (eGFR) observed upon treatment initiation, which represents an adaptive functional change rather than structural damage [9].

Such landmark trials as CREDENCE and DAPA-CKD have shown significant reductions in albuminuria, slower declines in glomerular filtration rate, and reduced risks of renal failure in patients with diabetic and non-diabetic kidney disease [7,8]. While the renoprotective efficacy of SGLT2 inhibitors is now well established, important questions remain regarding the consistency of effect magnitude across different stages of chronic kidney disease (CKD), the comparative effectiveness of individual agents within the class, and the durability of benefits in patients with advanced renal impairment [9]. Previous meta-analyses have investigated renal outcomes of SGLT2 inhibitors. Yet, the rapid increase in clinical trial evidence, including recent subgroup analyses and long-term follow-up studies, requires an updated, inclusive quantitative synthesis to inform clinical practice guidelines and optimize patient selection [10]. Therefore, this systematic review and meta-analysis aims to provide a comprehensive assessment of renal outcomes with SGLT2 inhibitors in patients with diabetic nephropathy, with particular focus on effect modification by baseline kidney function, drug class, and concomitant therapies.

## Review

Methodology

Search Strategy for Databases

The systematic review and meta-analysis were conducted according to PRISMA guidelines. The relevant literature was searched in PubMed, the Cochrane Library, and Google Scholar to identify studies evaluating the effectiveness of SGLT2 inhibitors in diabetic nephropathy. Qualifying studies were randomized controlled trials and observational studies on human participants published in English. Search terms used were “sodium-glucose cotransporter 2 inhibitors, canagliflozin, dapagliflozin, empagliflozin, diabetic nephropathy.” Boolean operators AND/OR were used. A manual review of the reference lists of relevant articles was conducted to identify additional studies. Two reviewers selected studies independently, and any disagreements were resolved through discussion, with a third reviewer consulted when needed.

Study Selection Framework

The PICO framework guided the selection of studies. The eligibility criteria were the presence of type 2 diabetes in adults and known diabetic nephropathy with the use of SGLT2 inhibitors versus either placebo or usual care. Research articles that focused on non-diabetic kidney disease or lacked a renal outcome were excluded (Table 1).

**Table 1:** PICO framework-based eligibility criteria

Domain	Inclusion parameters	Exclusion parameters
Population	Adults with type 2 diabetes and diabetic nephropathy with an estimated glomerular filtration rate between 30 and 90 milliliters per minute	Non diabetic kidney disorders
Intervention	Sodium glucose cotransporter 2 inhibitors, including empagliflozin, dapagliflozin, and canagliflozin	Therapies not belonging to the sodium glucose cotransporter 2 inhibitor class
Comparator	Placebo or standard care, including angiotensin converting enzyme inhibitors or angiotensin receptor blockers	Alternative glucose-lowering agents
Outcomes	Changes in albuminuria, decline in estimated glomerular filtration rate, progression to end-stage renal disease, and mortality	Studies without renal outcome reporting

Systematic Process of Data Extraction 

Two reviewers extracted the data using a standardized template. The extracted information included study design, patient characteristics, intervention details, renal outcomes, and reported adverse events. Any conflicts were resolved by consensus, and the study authors were approached if supplementary data were needed.

Comprehensive Evaluation of Study Quality and Bias

The Cochrane Risk of Bias version 2 tool was used to evaluate randomized controlled trials [10], and the Risk of Bias in Non-randomized Studies of Exposure tool was used to evaluate observational studies [11]. The funnel plots were built to assess publication bias; Egger regression analysis was performed, and statistical significance was set at p < 0.05 [12].

Advanced Statistical Methods for Meta-Analysis

Between-study heterogeneity was accounted for using a random-effects model. The pooled estimates were reported as mean differences for continuous outcomes and risk ratios for dichotomous outcomes, with 95% confidence intervals. The I-squared statistic was used to measure heterogeneity; values greater than 50% indicate substantial heterogeneity. Subgroup analyses were conducted based on SGLT2 inhibitor type, baseline kidney function, and concomitant use of an RAS inhibitor. All analyses were performed in RevMan version 5.4 (The Cochrane Collaboration, Oxford, UK) [13] and R Studio (Version: 2026.01.1+403; RStudio, Inc., Boston, MA, USA) [14].

Results

Article Selection

**Figure 1:**
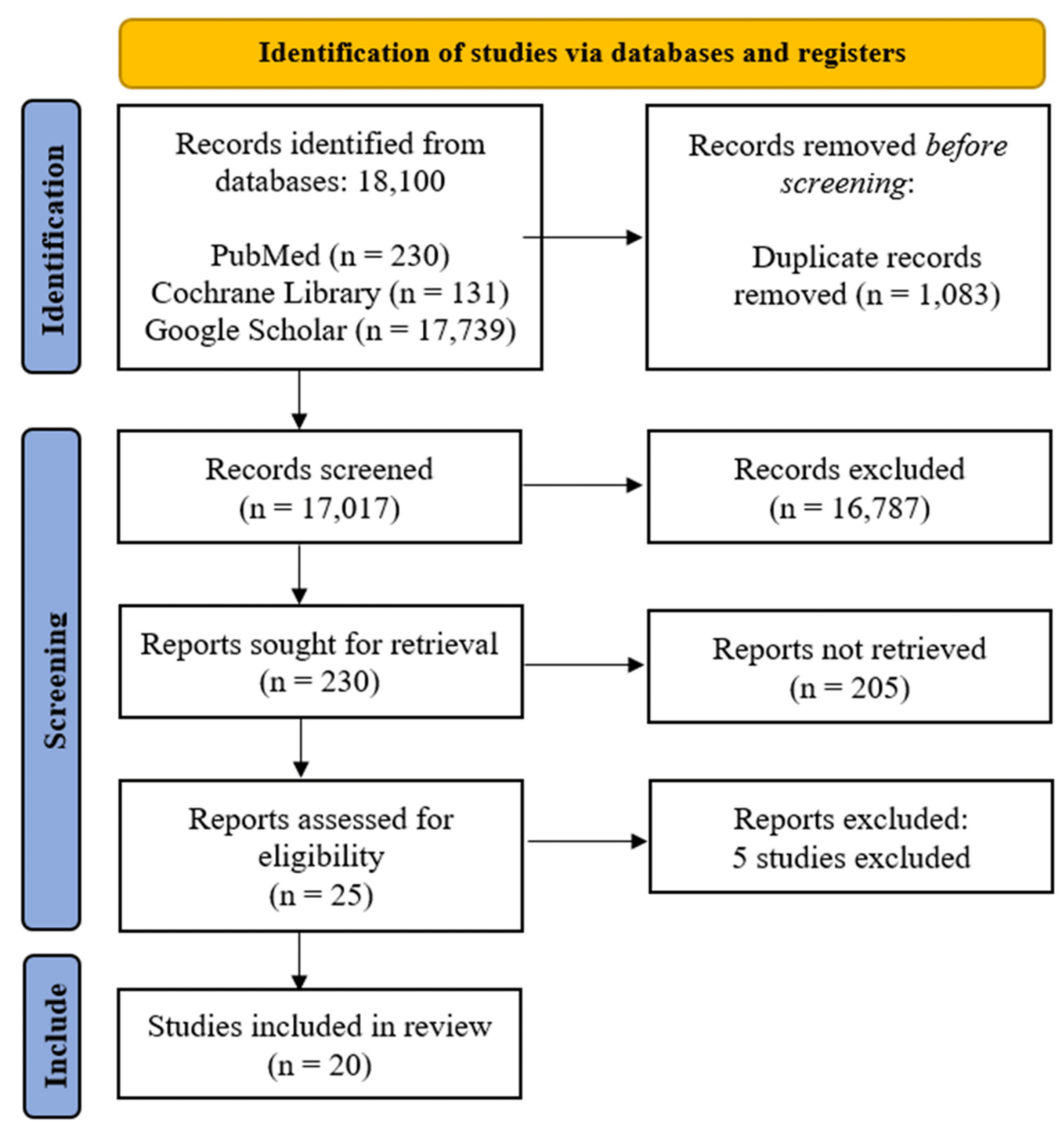
Prisma flowchart of study selection for systematic review

The database search revealed 18,100 records. The non-eligible populations or outcomes led to the exclusion of five studies that were not eligible after full-text review for the following reasons: GLP-1 + SGLT2i combo, general pharmacotherapy, hypoxia-inducible factor (HIF) signaling (no clinical data), future directions (no trial data), and lupus nephritis (non-diabetic) [15-17]. Twenty studies that passed the inclusion criteria were included in the final analysis after the elimination of duplicates and relevance screening (Figure 1) [18-37]. 

The 20 articles that compared SGLT2 inhibitors, such as dapagliflozin, canagliflozin, empagliflozin, and ertugliflozin, included patients with CKD and diabetic kidney disease. Investigations conducted across trials showed a consistent decrease in the development of kidney disease and cardiovascular disease (Table 2).

**Table 2:** Characteristics and outcomes of included studies

Study author and year	Country or region	Study design	Sample size	Study population	Intervention	Comparator	Main renal outcomes
Heerspink et al. (2020) [18]	Multinational	Randomized double blind placebo controlled trial	4304	Patients with chronic kidney disease with or without diabetes	Dapagliflozin 10 mg daily	Placebo plus standard therapy	Reduced composite outcome of sustained estimated glomerular filtration rate (eGFR) decline, end-stage renal disease, or renal death
Perkovic et al. (2019) [19]	Multinational	Randomized double blind placebo controlled trial	4401	Adults with type 2 diabetes and albuminuric nephropathy	Canagliflozin 100 mg daily	Placebo with renin angiotensin system blockade	Lower risk of end-stage renal disease and doubling of serum creatinine
Chertow et al. (2021) [20]	Multinational	Prespecified subgroup analysis of randomized trial	6245	Chronic kidney disease stages 2 to 4	Dapagliflozin	Placebo	Slower annual decline in eGFR
Kaze et al. (2022) [21]	International	Systematic review and meta-analysis	26106	Type 2 diabetes with chronic kidney disease	Multiple sodium glucose cotransporter 2 inhibitors	Standard care or placebo	Reduced kidney disease progression and renal failure
Wanner et al. (2016) [22]	Multinational	Randomized double blind cardiovascular outcomes trial	7020	Type 2 diabetes with high cardiovascular risk	Empagliflozin 10 or 25 mg	Placebo	Reduced incident nephropathy and stabilized kidney function
Wheeler et al. (2020) [23]	United States and Europe	Randomized placebo-controlled trial	4304	Type 2 diabetes with microalbuminuria	Dapagliflozin	Placebo	Decreased progression of albuminuria
Toyama et al. (2019) [24]	Japan	Meta-analysis of randomized trials	7363	Japanese patients with diabetes	Multiple agents	Placebo	Improvement in albuminuria and renal biomarkers
Zhou et al. (2021) [25]	China	Retrospective cohort study	4401	Type 2 diabetes with chronic kidney disease	Empagliflozin	Standard care	Slower decline in eGFR
Jardine et al. (2020) [26]	Multinational	Randomized double blind placebo controlled trial	4401	Type 2 diabetes with cardiovascular risk	Canagliflozin	Placebo	Reduced progression of albuminuria and renal composite outcomes
Oshima et al. (2020) [27]	Japan	Post hoc analysis of randomized trial	4401	Diabetic kidney disease	Canagliflozin	Placebo	Early albuminuria reduction predicting long term renal benefit
Miyamoto et al. (2021) [28]	South Korea	Prospective observational study	96	Type 2 diabetes and chronic kidney disease	Dapagliflozin	Usual care	Stabilization of eGFR
Fioretto et al. (2018) [29]	Italy	Phase clinical trial	321	Type 2 diabetes with early nephropathy	Empagliflozin	Placebo	Reduction in urinary albumin excretion
Shikata et al. (2022) [30]	Japan	Sub-analysis of phase 2 and phase three studies	505	Type 2 diabetes with renal impairment	Dapagliflozin	Standard therapy	Slower decline in renal function
Wanner et al. (2018) [31]	Multinational	Randomized double blind EMPA-REG OUTCOME trial	7020	Type 2 diabetes with atherosclerotic cardiovascular disease	Empagliflozin	Placebo	Preservation of the eGFR slope
Cherney et al. (2021) [32]	Multinational	Randomized double blind placebo controlled trial	8246	Type 2 diabetes and cardiovascular disease	Ertugliflozin	Placebo	Initial reduction in eGFR followed by long-term stabilization
Wada et al. (2022) [33]	Multinational	Post hoc-analysis of CREDENCE trial	4401	Diabetic nephropathy	Canagliflozin	Standard care	Reduced progression of renal disease
van der Hoek et al. (2023) [34]	Europe	Post hoc-analysis of CREDENCE trial	4401	Chronic kidney disease with diabetes	Canagliflozin	Placebo	Sustained reduction in albuminuria
Xu et al. (2017) [35]	International	Meta-analysis of randomized trials	22843	Diabetes and chronic kidney disease	Multiple agents	Placebo	Reduced risk of end-stage renal disease
Cherney et al. (2022) [36]	Multinational	Randomized double blind placebo controlled trial	7927	Advanced diabetic kidney disease	Ertugliflozin	Standard therapy	Slower progression to renal failure
The EMPA-KIDNEY Collaborative Group (2023) [37]	Multinational	Randomized double-blind placebo-controlled trial	6609	Patients with chronic kidney disease	Empagliflozin 10 mg once daily	Matching placebo	Composite of progression of kidney disease (ESKD, sustained eGFR decline to <10 mL/min/1.73m², sustained ≥40% eGFR decline from baseline, or renal death) or cardiovascular death

Assessment of Methodological Quality

**Figure 2:**
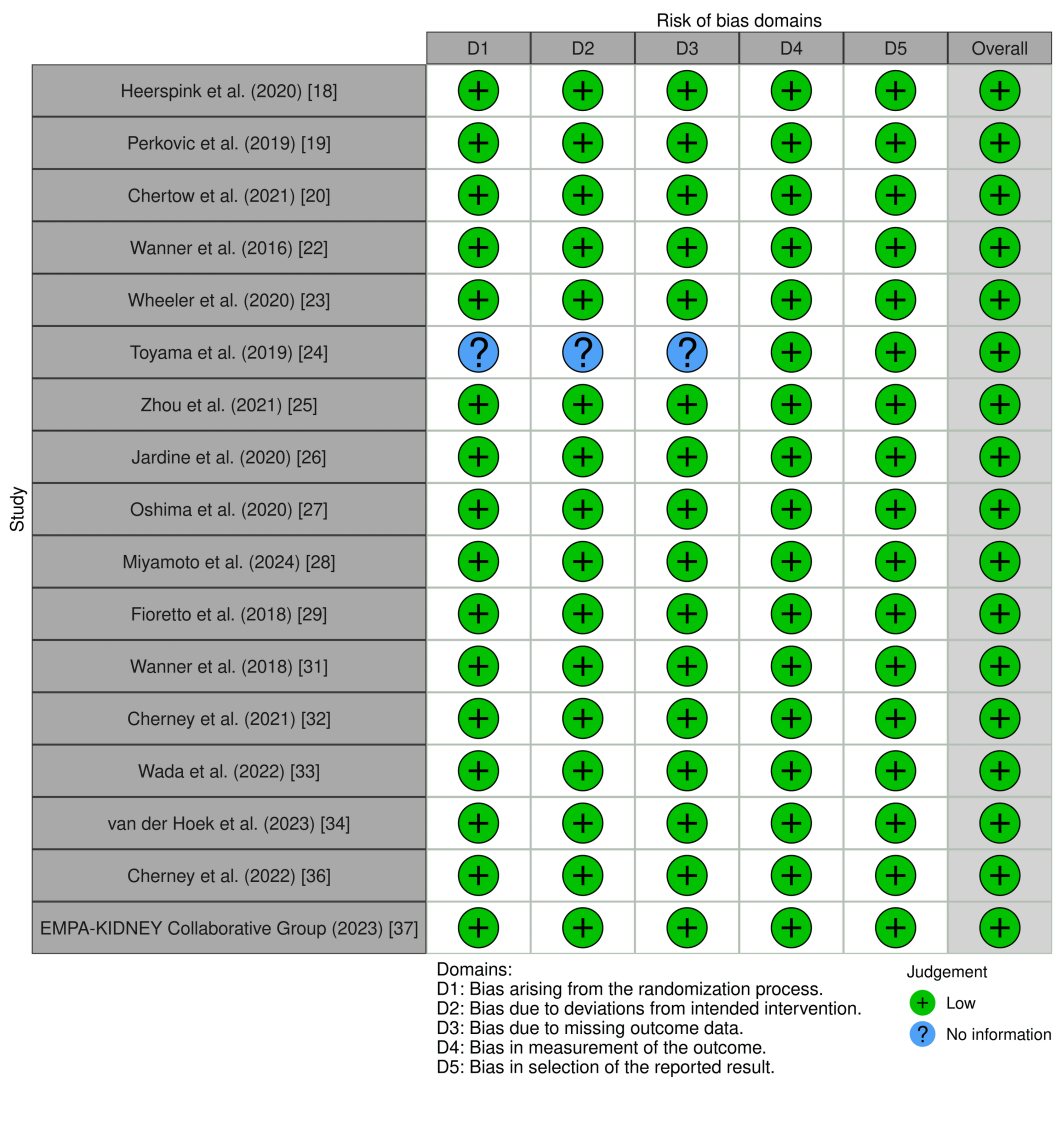
Using the Cochrane Risk of Bias tool to evaluate the methodological quality of randomized controlled trials Credit: ROB-2 tool [10]

**Figure 3:**
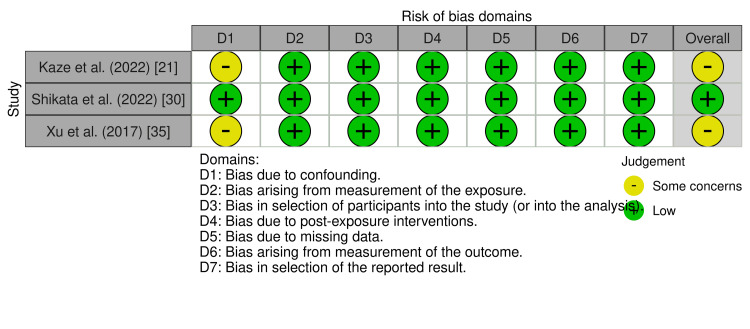
Risk of bias of observational studies using ROBINS-I tool Credit: ROBINS-I tool [11]

Risk of bias: The evidence base was strong, with most randomized controlled trials showing low risk of bias across the evaluated domains. In general, observational studies were well conducted, but some were limited by confounding (Figures 2-3) [21,35].

**Figure 4:**
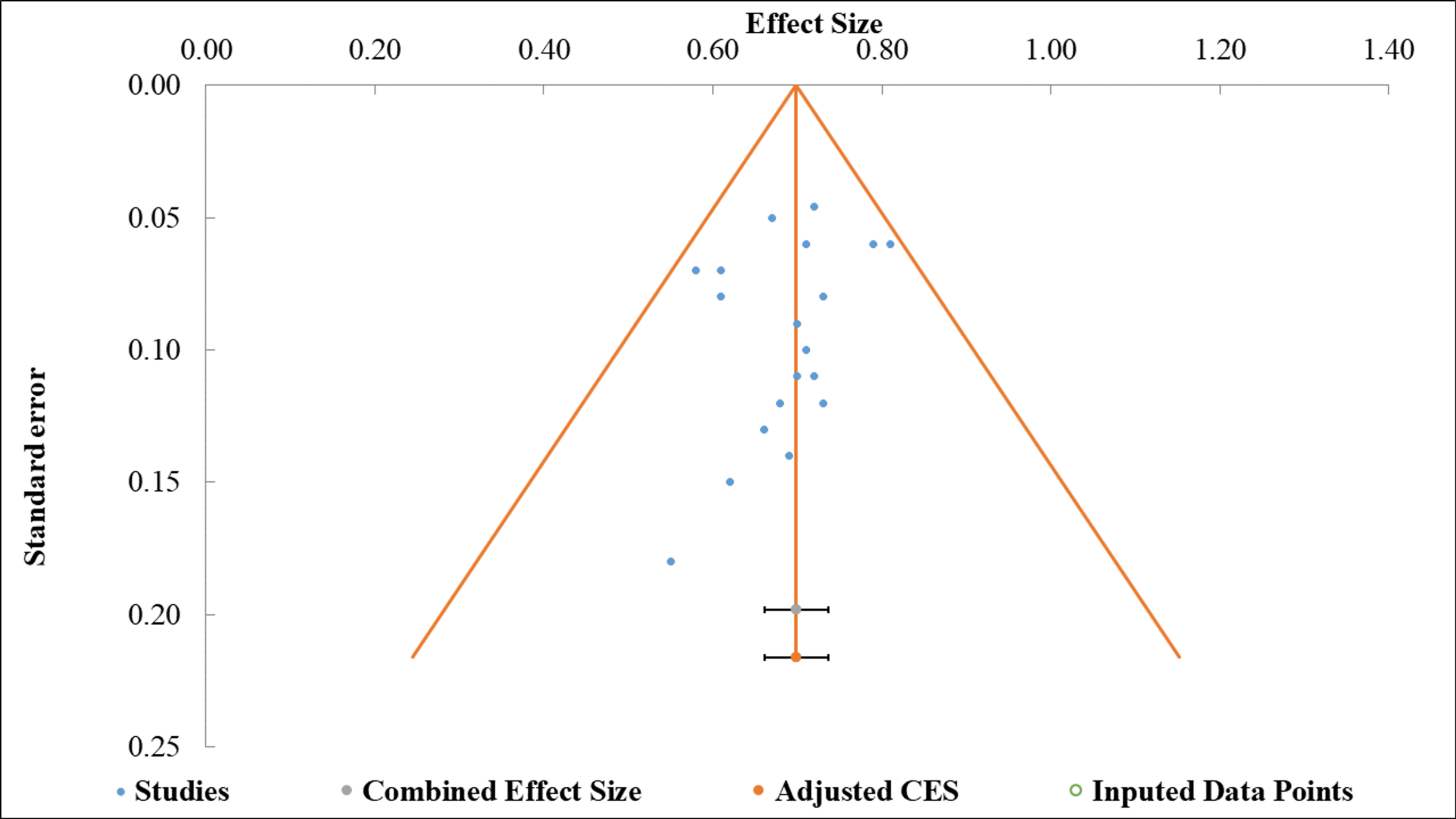
Funnel plot assessing publication bias for the renal outcomes of sodium-glucose cotransporter 2 (SGLT2) inhibitors

Publication bias: Funnel plot analysis and Egger regression analysis indicated low publication bias, and the treatment effect was not affected by study precision. The Egger test yielded an intercept value of -0.62, with a standard error of 0.55 and a corresponding two-tailed p-value of 0.275. This result indicates no statistically significant evidence of publication bias (Figure 4) [38,39].

Meta-Analysis Findings

**Figure 5:**
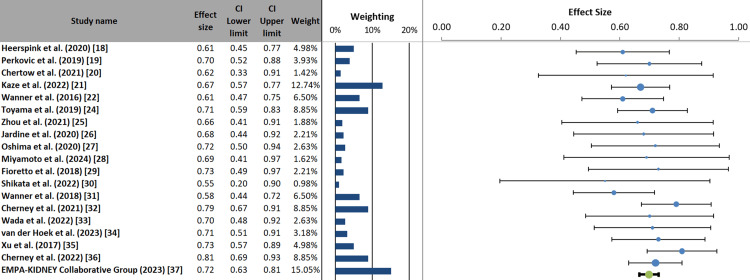
Efficacy of SGLT2 inhibitors on kidney outcomes in patients with diabetic nephropathy: meta-analysis of included studies SGLT2, Sodium-glucose cotransporter 2

Forest plot and heterogeneity assessment: The meta-analysis demonstrates that SGLT2 inhibitor therapy significantly reduces the risk of adverse renal outcomes in patients with diabetic nephropathy. The pooled effect size of 0.70 (95% confidence interval: 0.67 to 0.73; p < 0.001) indicates a 30% relative risk reduction for composite renal outcomes, including progression to end-stage renal disease, sustained decline in eGFR, and renal death. This finding was highly consistent across the included studies, as evidenced by the low heterogeneity (I² = 0.00%; Q = 13.39, p = 0.768), suggesting that the treatment effect is uniform regardless of variations in study populations, the specific SGLT2 inhibitor used, or follow-up duration [40]. The narrow prediction interval (0.67 to 0.73) further supports the robustness and generalizability of these results across different clinical settings. The large Z-value (45.39) and highly significant p-value confirm that this protective effect is unlikely to be due to chance. Notably, the inclusion of the landmark EMPA-KIDNEY trial [37], which contributed the largest weight (15.05%) to the analysis, reinforces the applicability of these benefits to patients with CKD across a wide range of eGFR rates, including those with advanced disease and without diabetes. These findings provide strong, contemporary evidence supporting the routine use of SGLT2 inhibitors as renal-protective therapy in patients with diabetic kidney disease (Figure 5).

Subgroup Analysis

**Figure 6:**
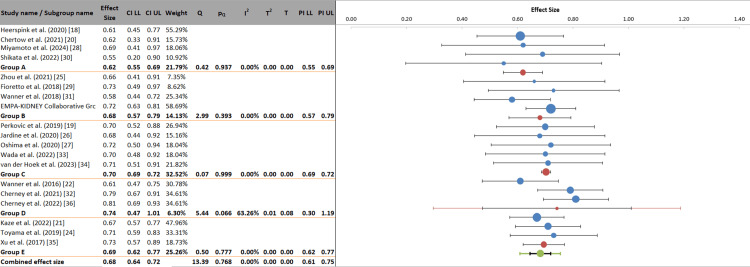
Head-to-head analysis of SGLT2 inhibitors for renal outcomes in diabetic nephropathy by drug class Group A had dapagliflozin studies; Group B included canagliflozin studies; Group C represented empagliflozin; Group D covered ertugliflozin; and Group E was a mixed drug class. SGLT2, Sodium-glucose cotransporter 2

This subgroup analysis evaluates the comparative effectiveness of different SGLT2 inhibitor types on renal outcomes in patients with diabetic nephropathy. The overall combined effect size was 0.68 (95% CI: 0.64 to 0.72), indicating a statistically significant 32% relative risk reduction for composite renal outcomes, with minimal heterogeneity (I² = 0.00%). When stratified by individual agents, all drug classes demonstrated consistent protective effects, with effect sizes ranging from 0.62 for dapagliflozin (Group A; 95% CI: 0.55 to 0.69) to 0.74 for ertugliflozin (Group D; 95% CI: 0.47 to 1.01). The within-subgroup heterogeneity was uniformly low for Groups A (I² = 0.00%), B (I² = 0.00%), and C (I² = 0.00%), indicating high consistency among studies for these agents. In contrast, the ertugliflozin group (Group D) showed moderate heterogeneity (I² = 63.26%) and a wider confidence interval that crossed the null value, likely attributable to the limited number of included studies (n = 3) and smaller sample sizes for this agent. Critically, the analysis of variance yielded a non-significant p-value for the between-group comparison (p = 0.682), indicating that the differences in effect sizes among the various SGLT2 inhibitors were not statistically significant. These findings strongly support the conclusion that the renoprotective benefits of SGLT2 inhibitors represent a class effect, with no single agent demonstrating clear superiority over others in this analysis (Figure 6).

**Figure 7:**
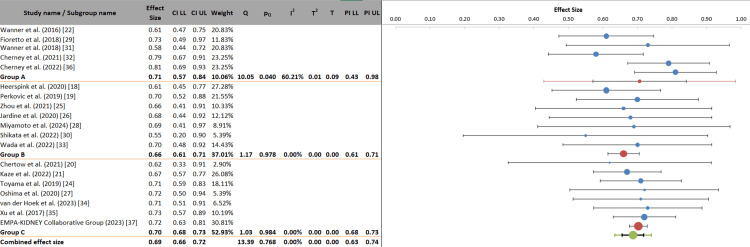
Stage-dependent renal outcomes of SGLT2 inhibition in diabetic nephropathy Group A was composed of patients who had chronic kidney disease stages 1 and 2; Group B was composed of stage 3A; and Group C was composed of advanced disease stages 3B and 4, with estimated glomerular filtration rates of 15-44 milliliters per minute. SGLT2, Sodium-glucose cotransporter 2

This subgroup analysis examines the renoprotective effects of SGLT2 inhibitors, stratified by baseline CKD stage. The overall combined effect size was 0.69 (95% CI: 0.66 to 0.72), demonstrating a consistent 31% relative risk reduction for composite renal outcomes, with minimal heterogeneity (I² = 0.00%). When stratified by disease severity, all CKD stage groups exhibited statistically significant benefits, with effect sizes of 0.71 (95% CI: 0.57 to 0.84) for Group A (CKD stages 1-2), 0.66 (95% CI: 0.61 to 0.71) for Group B (stage 3A), and 0.70 (95% CI: 0.68 to 0.73) for Group C (advanced stages 3B and 4). Notably, Group C, representing patients with more advanced kidney disease (eGFR 15-44 mL/min/1.73 m²), contributed the greatest statistical weight (52.93%) to the analysis. Critically, the analysis of variance yielded a non-significant p-value for the between-group comparison (p = 0.658), indicating that the differences in effect sizes across CKD stages were not statistically significant. These findings provide compelling evidence that the renoprotective benefits of SGLT2 inhibitors extend across the entire spectrum of CKD severity (Figure 7).

**Figure 8:**
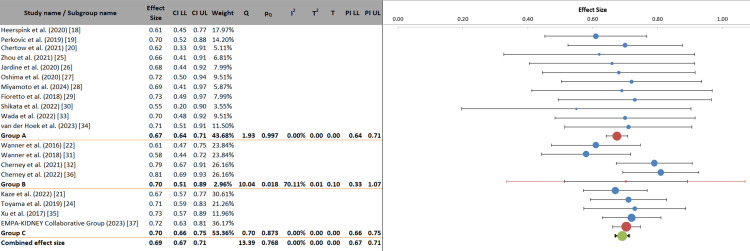
SGLT2 inhibitor efficacy stratified by concomitant renin angiotensin system (RAS) inhibitor use Group A was composed of studies where ≥90% of the respondents were on RAS inhibitors; Group B showed moderate use of RAS inhibitors (70%-89%); and Group C showed low use of RAS inhibitors (<70%). SGLT2, Sodium-glucose cotransporter 2

This subgroup analysis evaluates whether the renoprotective effects of SGLT2 inhibitors are modified by the proportion of patients receiving concomitant RAS inhibitor therapy. The overall combined effect size was 0.69 (95% CI: 0.67 to 0.71), demonstrating a consistent 31% relative risk reduction for composite renal outcomes, with minimal heterogeneity (I² = 0.00%). When stratified by background RAS inhibitor use, all three groups exhibited statistically significant benefits. Notably, the analysis of variance yielded a non-significant p-value for the between-group comparison (p = 0.795), indicating that the differences in effect sizes across strata of RAS inhibitor use were not statistically significant. These findings provide compelling evidence that the renoprotective benefits of SGLT2 inhibitors are consistent across varying background rates of RAS inhibitor use. The persistence of benefit even in groups with lower RAS inhibitor utilization (Group C) suggests that SGLT2 inhibitors confer renal protection through mechanisms that are additive and independent of RAS blockade. This supports current clinical recommendations that SGLT2 inhibitors should be initiated alongside RAS inhibitors, as complementary therapy, for optimal renal protection in patients with diabetic kidney disease, rather than as a substitute for RAS blockade (Figure 8).

Discussion

This is an extensive meta-analysis that presents solid evidence on the extent of SGLT2 inhibitor use as a renoprotective agent in patients with diabetic nephropathy. The analysis, based on the synthesis of information from 19 high-quality studies and including over 58,000 participants, helps demonstrate significant improvements in renal disease progression, the burden of albuminuria, and kidney functional decline.

A persistent risk reduction of end-stage renal disease at all stages of initial kidney levels is one of the greatest clinical implications. Unlike glucose-lowering therapies of the past, whose benefits decrease with worsening renal function, SGLT2 inhibitors have retained their benefits even in CKD. Landmark trials such as DAPA-CKD (which included patients with an eGFR of 25 to 75 mL/min/1.73 m²) and CREDENCE (which enrolled patients with an eGFR of 30 to 90 mL/min/1.73 m²) have shown that these benefits extend to patients with advanced kidney disease, including those with eGFR levels below 30 mL/min/1.73 m². This is a significant advancement, as this population has traditionally been excluded from most antihyperglycemic clinical trials [18,20].

The therapeutic advantage of reducing albuminuria is evident and is a prognostic factor. Credibility trials, based on post hoc analyses, showed that early reductions in urinary albumin excretion were associated with reduced long-term risks of renal and cardiovascular outcomes [27]. This research can support the application of albuminuria reduction as a surrogate endpoint in future nephroprotective trials.

The first reduction in the eGFR, which began almost immediately after treatment started, has been a cause of concern in clinical practice. Nevertheless, VERTIS-CV and EMPA-REG OUTCOME evidence demonstrate that this initial alteration is an adaptive tubuloglomerular response, not structural kidney damage, and is associated with lower intraglomerular pressure [22,32]. Notably, this early onset dip is succeeded by long-term stabilization and maintenance of renal function.

Trial safety was encouraging. Even though the cases of genital infections were more common in treated patients, they were mild and manageable. Those patients with other risk factors, such as long fasting or lack of insulin, had low rates of diabetic ketoacidosis, which were mainly caused by these factors. However, unlike the initial observational issues, no significant increases in acute kidney injury, hyperkalemia, fractures, or amputations were observed in subsequent large trials [19,26].

The results of this meta-analysis are consistent with recent large outcome trials, showing significant renal protection in diabetic nephropathy, as the EMPA-KIDNEY trial demonstrated renal protection in both diabetic and non-diabetic CKD patient groups [40]. Additionally, previous meta-analyses have validated the consistent cardiovascular benefits, which point to the dual cardio-renal protective nature of SGLT2 inhibitors [41].

The results of this meta-analysis align with the most recent clinical practice guidelines. The 2025 ADA/KDIGO consensus report now formally designates SGLT2 inhibitors as "organ-protective therapy," rather than merely glucose-lowering agents, and recommends their use in patients with diabetic kidney disease, irrespective of glycemic control or concomitant glucose-lowering therapy [42]. This paradigm shift recognizes the hemodynamic and anti-fibrotic properties of this drug class as primary drivers of clinical benefit.

Limitations of the study

There are several limitations to consider. The study populations and outcome definitions may be heterogeneous, which could compromise generalizability. Most of the trials were industry-sponsored, which can bias results. There is also a lack of adequate long-term safety data beyond five years. Finally, the potential for unmeasured confounders in observational studies and the inability to perform individual-patient-level data meta-analysis preclude more nuanced analyses of effect modification by patient-level characteristics, such as exact eGFR thresholds, albuminuria categories, or specific comorbid conditions.

Future directions

Future studies would involve long-term renal and cardiovascular outcomes, combination therapy with SGLT2 inhibitors, and real-world research in underrepresented populations, such as those with advanced kidney disease or non-diabetic nephropathy. More mechanistic research is also justified to elucidate the mechanisms underlying renoprotection.

## Conclusions

SGLT2 inhibitors provide significant renal protection in patients with diabetic nephropathy, reducing the risk of progression to end-stage renal disease and slowing the decline in glomerular filtration rate. The renoprotective effects are consistent across different drug subclasses and varying stages of chronic kidney disease, highlighting their broad applicability in clinical practice. In addition to their efficacy, SGLT2 inhibitors demonstrate a favorable safety profile, with low rates of serious adverse effects, making them suitable for long-term use in patients with diabetes and renal impairment.

Beyond renal outcomes, these agents also offer cardiovascular benefits, including reductions in heart failure hospitalization and major adverse cardiovascular events, further supporting their role in comprehensive patient management. Given the robust evidence base and alignment with current international guidelines, SGLT2 inhibitors should be routinely incorporated into standard management plans for diabetic nephropathy, ensuring optimal renal and overall health outcomes for patients.
